# The HMG-box protein PtHMG1 modulates lipid accumulation and fatty acid composition in the diatom *Phaeodactylum tricornutum*

**DOI:** 10.3389/fpls.2026.1863846

**Published:** 2026-08-06

**Authors:** Yuanfeng Li, Ning Ma, Pu Song, Shaokun Dong, Ronglu Liu, Youyi Zhou, Hongjin Qiao

**Affiliations:** 1 School of Life Science, Ludong University, Yantai, China; 2 Yantai Microalgae Engineering Technology Research Center, Yantai, China

**Keywords:** desaturase, metabolic regulation, *Phaeodactylum tricornutum*, polyunsaturated fatty acids, transcriptional regulator

## Abstract

**Introduction:**

The diatom *Phaeodactylum tricornutum* is a premier biological platform for producing high-value polyunsaturated fatty acids (PUFAs). However, the transcriptional mechanisms coordinating lipid accumulation and fatty acid composition remain poorly understood.

**Methods:**

In this study, we characterized the role of the HMG-box transcriptional regulator *PtHMG1* in *P. tricornutum* through overexpression (OE) and CRISPR/Cas9-mediated knockout (KO).

**Results:**

Our results identify *PtHMG1* as a global regulator of lipid metabolism. PtHMG1-OE strains exhibited a 90% increase in total fatty acids and a rise in total lipids from 0.3 g g^-1^ to 0.5 g g^-1^ without significant growth inhibition. Although OE upregulated key front-end desaturases (PTD5a, PTD5b, and PTD6) and increased absolute eicosapentaenoic acid (EPA) content by 18%, the relative proportion of PUFAs declined due to the disproportionate accumulation of saturated and monounsaturated fatty acids. Conversely, PtHMG1-KO led to an 11% decrease in total lipids and significant growth defects. While KO strains showed downregulation of EPA-specific desaturases, a compensatory upregulation of alternative desaturases maintained a stable relative fatty acid profile.

**Conclusion:**

These findings demonstrate that *PtHMG1* plays a crucial role in regulating total lipid accumulation and modifying fatty acid composition in *P. tricornutum*. The decoupling of lipid enhancement from growth inhibition underscores the significant potential of utilizing *PtHMG1* for targeted genetic modification, facilitating the sustainable development of high-yield microalgal oils.

## 1 Introduction

As indispensable components of the global ecosystem and the biogeochemical carbon cycle, diatoms drive approximately 40% of oceanic carbon fixation and 20% of Earth’s primary productivity. As foundational primary producers, they sustain diverse planktonic and benthic food webs (Field et al., 1998). Beyond their ecological significance, diatoms are recognized as a premier biological platform for producing high-value bioactive compounds, particularly long-chain polyunsaturated fatty acids (LC-PUFAs). These lipids possess significant pharmacological potential due to their anti-inflammatory, cardioprotective, and neuroprotective properties (Hildebrand et al., 2012). Among oleaginous diatoms, *Phaeodactylum tricornutum* has emerged as a model organism. Its fully sequenced genome, coupled with a robust genetic toolkit including CRISPR/Cas9-mediated gene editing and efficient transformation systems, enables the precise elucidation of complex metabolic and regulatory pathways (De Riso et al., 2009; Nymark et al., 2016; Wang et al., 2018).

The biosynthesis of long-chain PUFAs (LC-PUFAs) in *P. tricornutum* is a sophisticated, multi-step process characterized by a series of sequential desaturation and elongation reactions. This process typically initiates with the *de novo* synthesis of C16 and C18 saturated fatty acids (SFAs) in the plastid, which are then exported to the endoplasmic reticulum (ER) for further modification (Guschina and Harwood, 2006). In the ER, the “front-end” desaturation pathway represents the primary route for eicosapentaenoic acid (EPA) production. This pathway begins with the conversion of oleic acid (OA, C18:1n-9) into linoleic acid (LA, C18:2n-6) and α-linolenic acid (ALA, C18:3n-3). Subsequently, “front-end” desaturases (typically Δ6 and Δ5 desaturases) introduce double bonds at the carboxyl end (front end) of the fatty acid chain. Specifically, a Δ6-desaturase converts LA and ALA into γ-linolenic acid (GLA) and stearidonic acid (SDA), respectively. Following a C18-to-C20 elongation step, Δ5-desaturases catalyze the final desaturation to yield EPA (Arao et al., 1994; Kjær et al., 2016). The efficiency of these “front-end” enzymes is a critical determinant of the metabolic flux directed toward highly unsaturated fatty acids (HUFAs), as they must compete for a limited pool of acyl-CoA substrates (Meesapyodsuk and Qiu, 2012).

Despite the crucial role of these enzymes, current research has predominantly focused on the overexpression of individual desaturase genes or the optimization of environmental factors to boost EPA content. For instance, the plastidial Δ9 desaturase has been shown to influence both triglyceride (TAG) metabolism and EPA synthesis (Smith et al., 2021). Similarly, environmental stressors like salinity acclimation and fulvic acid treatment have been found to upregulate the expression of *PTD6*, *PTD5a*, and *PTD5b*, leading to significant EPA accumulation (Wang et al., 2019). Peng et al. (2014) further demonstrated that overexpressing *PTD5b* not only increased EPA content by 58% but also promoted the synthesis of upstream monounsaturated fatty acids (MUFAs), suggesting its role as a key regulatory node. However, the intrinsic mechanisms that govern the transcriptional orchestration of these desaturases remain largely unexplored.

Recently, the role of transcriptional regulators in algal lipid metabolism has received increasing attention. Members of the bZIP, Dof, and MYB families have been identified as pivotal regulators of lipid droplet formation and TAG biosynthesis in various microalgae (Bai et al., 2021; Salas-Montantes et al., 2018; Shi et al., 2022; Takahashi et al., 2021). In *P. tricornutum*, it is highly probable that the expression of desaturases is under the stringent control of specific TFs that respond to physiological demands. To identify these regulators, we previously conducted a comparative proteomic analysis under stress conditions (low temperature, DTT, and methyl jasmonate) known to induce PUFA accumulation. This screening yielded a pool of candidate regulatory proteins and potential transcriptional regulators. Through a subsequent preliminary screening and bioinformatic prioritization of these candidates, PtHMG1 (ID: 7199075), a protein containing a conserved HMG-box domain, emerged as a particularly compelling target for further investigation. HMG-box proteins represent a unique class of TFs characterized by their ability to bind the minor groove of DNA, inducing significant structural bending. This architectural role allows them to facilitate the assembly of multi-protein transcription complexes, thereby acting as “global tuners” for complex metabolic pathways rather than simple on/off switches. In terrestrial plants, HMG-box proteins are known to regulate stress responses and developmental transitions by reshaping the chromatin landscape (Antosch et al., 2012; Potters et al., 2010). Beyond plants, HMG-box proteins are widely conserved across eukaryotes as architectural regulators that induce significant DNA conformational changes, thereby facilitating the recruitment of additional transcription factors to orchestrate fundamental processes such as sex determination in mammals and reproductive specification in fungi (Luthringer et al., 2024). However, their potential involvement in microalgal lipid homeostasis remains an untapped area of research. This led us to hypothesize that *PtHMG1* serves as a high-level architectural regulator that coordinates the expression of desaturation pathways to balance lipid quantity and quality.

In this study, we employed both overexpression and CRISPR/Cas9-mediated knockout to characterize the functional role of *PtHMG1*. Our results demonstrate that while *PtHMG1* is a potent driver of total lipid productivity, its regulation of the desaturation landscape is complex, reflecting a sophisticated trade-off between saturated fatty acid accumulation and PUFA proportion. This work provides new insights into the transcriptional orchestration of diatom lipid metabolism and identifies a novel genetic target for the bioengineering of high-value microalgal oils.

## 2 Materials and methods

### 2.1 Algal cultures and growth conditions

The wild-type (WT) strain *P. tricornutum* CCAP 1055/1 was sourced from the Culture Collection of Algae and Protozoa (CCAP). Algae were purified using f/2 solid medium with 1% agarose (Guillard and Ryther, 1962), and after the algal colonies grew, they were moved to the liquid medium for additional incubation. All subsequent physiological characterization and growth comparative experiments between the WT control and the mutant lines were conducted under strictly identical environmental conditions using the liquid f/2 medium. For these comparative assays, cultures were maintained in 500 mL Erlenmeyer flasks filled with 300 mL of f/2 medium with an initial inoculation density of 5×10^5^ cells mL^−1^. All parallel cultivations were incubated at 20 ± 0.5°C under a 12:12 h light:dark cycle with a light intensity of 75 μmol m^−2^ s ^−1^ and continuous shaking at 100 rpm. Each experimental group was performed in triplicate. Samples were collected daily for cell counting (Countstar IA1000, Shanghai RuiYu Biotech Co., Ltd., Shanghai, China). The growth rate (μ) was calculated based on cell concentration measurements, employing the equation (Orefice et al., 2019):


$$\;\text{μ}=({\text{lnN}}_{\text{t}}–{\text{lnN}}_{0})/\text{t}$$

where μ is the growth rate, N_0_ is the starting cell concentration, N_t_ is the cell concentration at the end day of the exponential growth phase, and t is the end day of the exponential growth phase.

Generally, algal cells were harvested on day 7 for subsequent transcriptional and biochemical analyses. This specific time point was selected based on our preliminary physiological evaluations, which established day 7 as the critical transition phase for the initiation of robust lipid accumulation in *P. tricornutum* (Qiao et al., 2016).

### 2.2 Bioinformatics analysis

Gene-specific primers were designed using Primer Premier 5 to amplify the complete length coding sequence (CDS) of *PtHMG1* (accession number: XM_002185227) based on the NCBI database. The accuracy of the *PtHMG1* sequence was verified by sequencing the amplicons. To identify homologs of PtHMG1, a blastp search was performed using the PtHMG1 protein sequence as a query. The amino acid sequences of the PtHMG1 homologs were aligned using MAFFT (http://mafft.cbrc.jp/alignment/software/windows_without_cygwin.html), and a maximum likelihood phylogenetic tree was constructed using MEGA 7.0 (Tamura et al., 2011) with 100 bootstrap replicates. Meanwhile, a comprehensive multiple sequence alignment was performed by comparing PtHMG1 with well-characterized HMG proteins from established model organisms. The 3D structure of PtHMG1 protein was predicted using the online tool AlphaFold (https://alphafold.ebi.ac.uk), and protein structure was edited with PyMOL software. To identify potential NLS motifs, three different algorithms were employed. Specifically, NLStradamus was used with a 2-state HMM static pre-loaded model and a prediction cutoff of 0.6 (Nguyen et al., 2009); DeepLoc-2.1 was run using the high-quality (Slow) model (Nielsen, 2025); and cNLS Mapper was utilized with a stringency cut-off score of 2.0 (Kosugi et al., 2009).

### 2.3 Construction of vectors for *PtHMG1* overexpression

The cDNA sequence of *PtHMG1* was obtained by reverse transcription from the RNA of *P. tricornutum*. The coding sequence (1029 bp) of the *PtHMG1* gene was amplified for overexpression using the primers *PtHMG1*-F and *PtHMG1*-R. Primers were designed using online website (https://tool.vazyme.com:18002/cetool/simple.html) (**Supplementary Table 1**). *PtHMG1* was placed to the downstream of fcpA promoter in plasmid pPha-T1 (Niu et al., 2012) (**Supplementary Figure 1**) after digestion with EcoR I and BamH I (Sangon Biotech. Co. Ltd, Shanghai, China) by using ClonExpress II One Step Cloning Kit (Vazyme Biotech Co. Ltd, Nanjing, China) (Zhang et al., 2023). The circularized plasmids were subsequently cloned into *Escherichia coli* TOP10 cells and incubated at 37°C overnight. After incubation, the cells were plated onto LB agar and cultured for an additional 12 hours. Positive clones were identified via colony PCR and submitted to Sangon Biotech. Co., Ltd. for sequencing. Positive strains were preserved in 50% glycerol for later use.

The recombinant vector pPha-T1-*PtHMG1* was introduced into wild-type *P. tricornutum* using biolistic transformation with the Biolistic PDS-1000/He Particle Delivery System (Bio-Rad, Hercules, CA, USA) (Falciatore et al., 1999; Krämer et al., 2022). To select the overexpression clones, the transformed cells were cultured on agar plates composed of 50% fresh seawater and 1% agar, supplemented with 100 μg mL^−1^ zeocin (Invitrogen, Carlsbad, CA, USA). These plates were incubated under an irradiance of 75 μmol photons m^−2^ s^−1^ for 2–3 weeks. Subsequently, resistant colonies were transferred to f/2 liquid medium containing 50 μg mL^−1^ zeocin for further cultivation.

### 2.4 Construction of *PtHMG1* knockout strains using CRISPR/Cas9

Using a previously established method, *PtHMG1* knockout strains were constructed by delivering the Cas9 system into *P. tricornutum* via plasmid conjugation from a bacterial donor cell (Nymark et al., 2017; Slattery et al., 2018). Briefly, sgRNAs targeting the positions 67–86 of the PtHMG1 open reading frame were designed on the website (http://crispor.tefor.net/), (**Supplementary Table 1**). The oligonucleotide designed to correspond to the predicted sgRNA binding site, along with its complementary counterpart, was synthesized, phosphorylated, and annealed. Subsequently, using the Golden Gate assembly method (New England Biolabs, Beverly, MA, USA), the cloned sgRNA was inserted into the pPtGE35 plasmid, which was procured from Addgene (107999) (**Supplementary Figure 2**). The Golden Gate reaction product was introduced into EPI300 *E. coli* cells via heat shock. The correctly cloned sgRNA plasmid was subsequently introduced into EPI300 *E. coli* cells that contained the pTA-Mob plasmid from Addgene (149662).

Subsequently, following an established protocol, the pPtGE35 plasmid containing sgRNA inserts was transferred into *P. tricornutum* through conjugation with *E. coli*. The screening for *PtHMG1* knockout in *P. tricornutum*, induced by Cas9, was conducted as previously described with minor modifications (Wang et al., 2018). The construction and screening of *P. tricornutum* knockout mutants were performed using optimized solid L1 medium (Foo et al., 2023). After a period of 2–3 weeks, exconjugants of *P. tricornutum* were randomly selected and inoculated into f/2 liquid medium supplemented with 50 μg mL^−1^ zeocin, and grown for 7 days. The knockout results were validated using T7 endonuclease assays, employing the PCR-specific primers PtHMG1-T7-F and PtHMG1-T7-R (**Supplementary Table 1**). The PCR products were subsequently subjected to Sanger sequencing to confirm the deletion length.

### 2.5 RNA extraction and quantitative reverse transcription PCR

Total RNA was extracted from *P. tricornutum* cells using the TRIzol method as previously described (Rio et al., 2010). Briefly, algal cells were harvested by centrifuging 5 mL of culture at 3500 g for 5 min at 4 °C. After discarding the supernatant, the pellet was rapidly frozen in liquid nitrogen, and the total RNA was extracted using TRIzol (Takara Bio, Otsu, Japan). Using a PrimeScript RT Reagent Kit with gDNA Eraser (Takara Bio, Otsu, Japan), the total RNA was converted into first-strand cDNA. qRT-PCR was performed as previously described (Feng et al., 2021). The products were detected using the Eppendorf Mastercycler ep realplex (Eppendorf AG, Hamburg, Germany). Specific primers used in this study were either adopted from validated literature sources or designed using online primer-design software (http://liuzhen106.com/tools/101_2.html), (**Supplementary Table 1**). The actin gene was used as the internal control (Song et al., 2023). Primer efficiencies were validated via standard curves and the data were processed using the 2^-ΔΔCt^ method.

### 2.6 Extraction and analysis of fatty acids

We analyzed the fatty acid composition as described in our previous publication (Qiao et al., 2015). On the seventh day of algal cell growth, around 0.1-0.5 g of algal cells were gathered by centrifuging and transferred to a screw-capped test tube measuring 12 cm × 1.5 cm. Five hundred microliters of toluene with the internal standard (0.1 mg glyceryl triheptadecanoate) and 1 mL of 0.5 M NaOH methanol solution were gradually added. After vortexing and mixing, the mixture was placed in a water bath at 80°C for 20 min, with constant shaking. Once cooled to room temperature, 1 mL of 10:100 (v/v) acetyl chloride in anhydrous methanol was added and the above warm bath step was repeated. After cooling the mixture to room temperature for 5 min, 1 mL of 6% K_2_CO_3_ and 400 μL of n-hexane was added, vortexed and mixed well, and the supernatant was separated by centrifuging at 4000 g for 1 min, with the upper layer being collected. The resulting fatty acid methyl esters were then subjected to analysis via gas chromatography (GC-1949; Panna, Changzhou, China) equipped with a fused silica capillary column (Supelco SP-2560: 100 m × 0.25 mm, film thickness 0.20 µm; Bellefonte, PA, USA). The heater was kept at 260°C using nitrogen as the carrier gas, while the column temperature increased from 140 to 260°C at a rate of 10°C per min. Fatty acids were identified by correlating the relative retention times with those of the reference standards (Merck, Rahway, NJ, USA). Fatty acid quantification was performed using two distinct metrics: absolute content and relative composition. The absolute content of individual fatty acids was determined by the internal standard method and normalized to the dry cell weight (expressed as mg g^-1^ DCW). The relative composition of each fatty acid component was calculated as the percentage of its individual peak area relative to the sum of all fatty acid peak areas, expressed as % of total fatty acids.

### 2.7 Total lipid content determination

Total lipid content was achieved using a modified procedure of Folch et al. (1957). Centrifuge tubes were pre-weighed (G1). On day 7 of growth phase, *P. tricornutum* culture was harvested by centrifugation (4,000 × g, 10 min, 4 °C). The resulting cell pellets were washed three times with 0.9% NaCl to remove residual medium. After carefully removing the moisture on the tube wall, the total weight (G2) was recorded. The biomass (G) was calculated using the following equation, where 0.125 is the predetermined ratio of biomass dry weight per gram of cell pellets.


G=(G2−G1)×0.125

The cell pellets were frozen at −80 °C overnight, vacuum freeze-dried, and ground into a fine powder. Then, frozen cells (approximately 20 mg) were extracted by adding methanol/chloroform (1:2 v/v). Following vortex mixing for 2 min, the mixture was centrifuged at 4,000 × g for 5 min. The resulting supernatant was collected in a new tube and a solution of sodium chloride (0.9%) was added in a proportion of 1:5 mL of lipid extract. The mixture was gently vortexed and allowed to stand for 5 min until two distinct phases were observed. The supernatant, which was rich in non-lipid components, was discarded. The oily phase was collected as the crude lipid extract. Following nitrogen evaporation, the residue was dried in an oven at 85 °C for 15 min and then weighed accurately. Total lipid content was expressed relative to the dry biomass weight.

### 2.8 Statistical analysis

Data analysis was performed using the software Origin 2024 and GraphPad Prism 9.5, and all experimental data were expressed as the mean ± standard deviation (SD) of three replicates. To evaluate the physiological and metabolic impact of each distinct genetic modification, pairwise statistical comparisons between each individual mutant line (OE or KO) and the corresponding WT control were executed using the two-tailed independent Student’s *t*-test. Significant differences were evaluated at the level of *P* < 0.05.

## 3 Results

### 3.1 Bioinformatics analysis of *PtHMG1*

**Figure 1:**
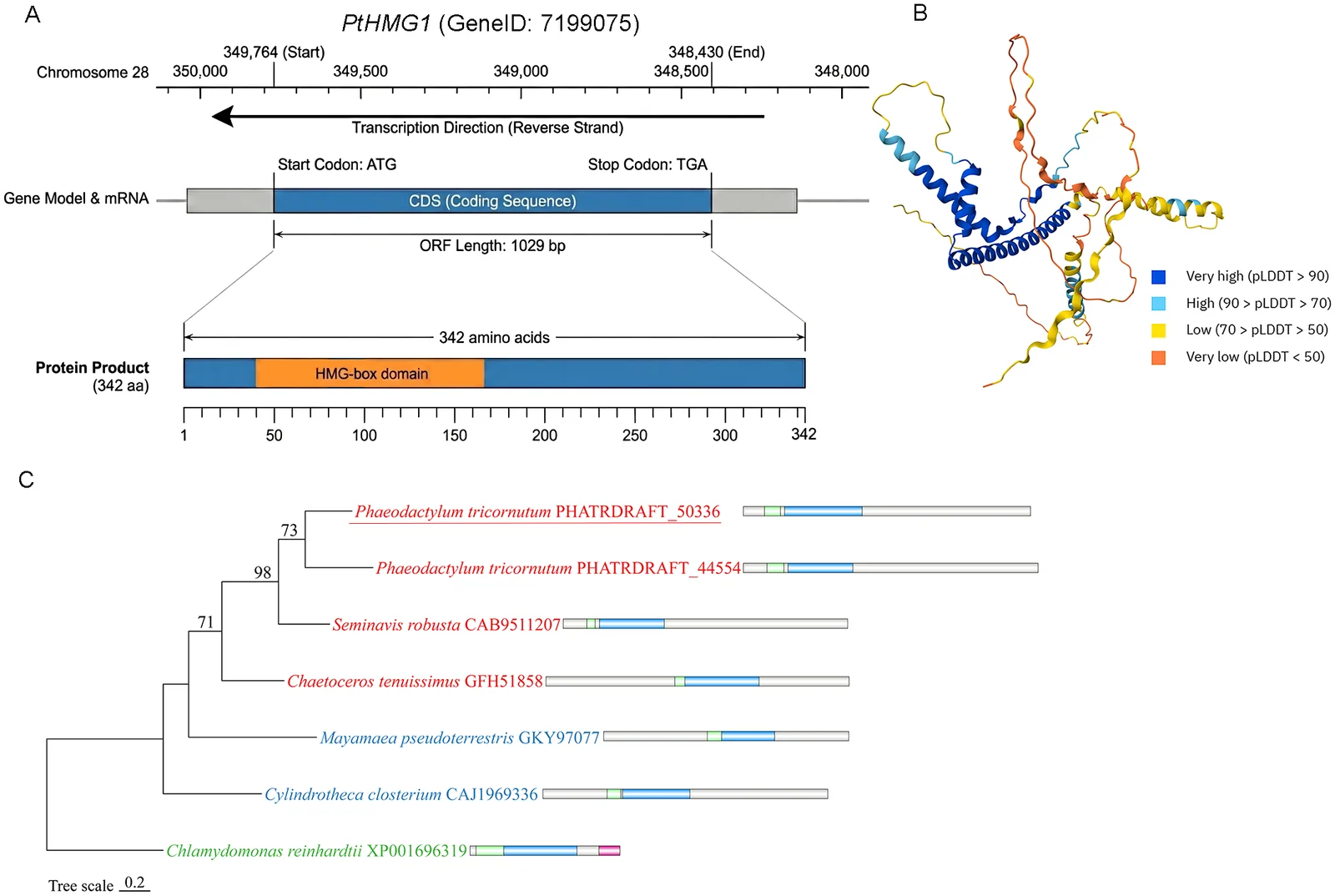
Bioinformatics analysis of *PtHMG1*. **(A)** Schematic drawing of the *PtHMG1* gene on chromosome 28, which is annotated as an open reading frame of 1029 bp in length corresponding to a predicted protein of 342 amino acids. **(B)** 3D structure of PtHMG1 with the local Distance Difference Test (pLDDT) scores. The blue area is the HMG-box domain. Image comes from AlphaFold. **(C)** Molecular phylogenetic analysis of HMG-box containing proteins among Bacillariophyta. Phylogenetic tree was constructed using the maximum likelihood method. Bootstrap values from 100 replicates were used to assess the robustness of the trees. The scale bar indicated 0.2 substitutions per site. The schematic of the protein structure was retrieved from UniProt, where green represents the N-terminal basic region, blue denotes the HMG-box domain, and red indicates the C-terminal acidic region.

The *PtHMG1* gene (Gene ID: 7199075) is located on chromosome 28 of *P. tricornutum* and encodes a protein consisting of 342 amino acids. This protein contains an HMG-box conserved domain spanning amino acids 49–164 at the N-terminus. The predicted gene structure and the 3D structure of the protein are depicted in **Figure 1**. The predicted structural model revealed a distinct modular organization characteristic of the High Mobility Group (HMG) superfamily. Crucially, the functional core of PtHMG1 is composed of a well-defined HMG-box domain, which adopts a canonical L-shaped topology stabilized by three closely packed α-helices. This structural core exhibited very high prediction confidence, with local Distance Difference Test (pLDDT) scores consistently exceeding 90. In sharp contrast, both the extensive N-terminal and C-terminal regions flanking the HMG-box core displayed very low pLDDT scores (less than 50) and lacked defined secondary structures, indicating a high degree of intrinsic disorder. The presence of these extended intrinsically disordered regions (IDRs) flanking a highly structured DNA-binding core strongly supports the role of PtHMG1 as a flexible transcriptional regulator capable of mediating dynamic macromolecular interactions.

To elucidate the evolutionary relationships and structural conservation of PtHMG1, a phylum-level phylogenetic tree was reconstructed with robust bootstrap support at each node (**Figure 1C**). The resulting topology demonstrated that PtHMG1 has a homolog protein PHATRDRAFT_44554 in *P. tricornutum* with a similarity of 48%. Both proteins clustered closely with *Seminavis robusta* (CAB9511207) with a strong bootstrap value of 98%, and with *Chaetoceros tenuissimus* (GFH51858) at a support value of 71%. These species collectively belong to the class Bacillariophyceae, reflecting the highly conserved distribution of this regulator among diatoms. Furthermore, *in silico* multiple sequence alignment with canonical HMG proteins from established model organisms indicated that PtHMG1 exhibits pronounced sequence homology to the HMGB sub-family (**Supplementary Figure 3**). Interestingly, while PtHMG1 and its diatom homologs are predicted to contain the characteristic core DNA-binding HMG-box domain, they conspicuously lack the acidic C-terminal tail that is typically prevalent in *Chlamydomonas reinhardtii* and higher eukaryotic HMGB proteins (**Figure 1C**; Antosch et al., 2012). This structural divergence strongly suggests that PtHMG1 may employ a distinct mechanism for architectural chromatin remodeling tailored specifically to diatoms. To investigate the potential nuclear localization of PtHMG1, we performed *in silico* analysis using three independent web-based platforms. As summarized in **Table 1**, NLStradamus, DeepLoc-2.1, and cNLS Mapper consistently predicted the presence of a nuclear localization signal. Specifically, NLStradamus and cNLS Mapper pinpointed a highly basic amino acid cluster located between residues 26 and 52, while DeepLoc-2.1 assigned a high probability (0.95) to its nuclear residency. This high degree of consensus across different algorithms strongly suggests that PtHMG1 serves as a potential intranuclear transcriptional regulator.

**Table 1:** *In silico* prediction of nuclear localization signals (NLS) in PtHMG1 using multiple algorithms.

Prediction tool	Predicted NLS sequence	Position	Score/probability
NLStradamus	-–-KPKTAKGKSSKTK KQKQYRAKKPKDMPRR	24–52	1.00 (Threshold: 0.6)
DeepLoc-2.1	ADGTSKPKTAKGKSSKTK KQKQYRAKKPKDMPRR	19-52	0.95 (Nucleus)
cNLS Mapper	-–KTAKGKSSKTK KQKQYRAKKPKDMPRR	26–52	2.10 (Cutoff: 2.0)
Consensus	-–KTAKGKSSKTK KQKQYRAKKPKDMPRR	26–52

### 3.2 Generation of transgenic *P. tricornutum* overexpressing *PtHMG1*

**Figure 2:**
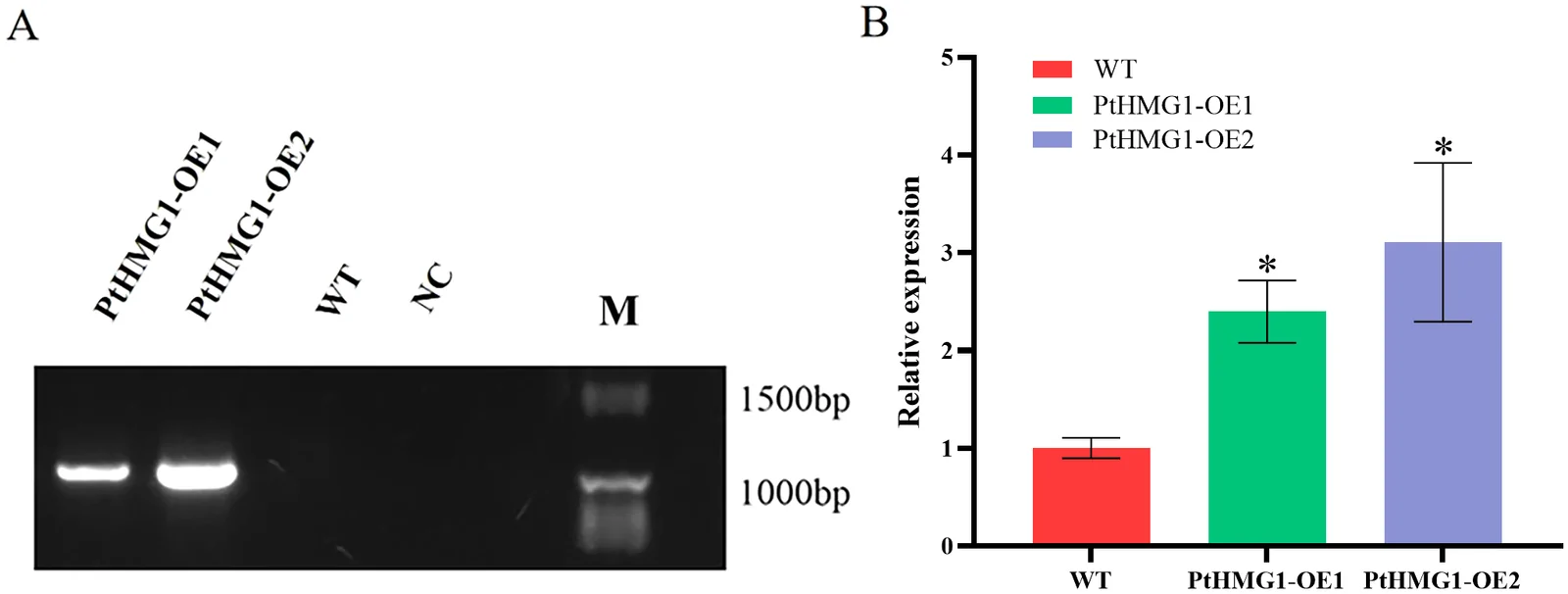
Generation of *PtHMG1* overexpressing (OE) strains. **(A)** Agarose gel of PCR products from genomic DNA in WT and PtHMG1-OE strains using the primers pPha-T1-F and PtHMG1-R. *PtHMG1-OE1* and *PtHMG1-OE2* are overexpressed algal strains, WT is wild type, NC is the blank control and M is Marker DL2000. **(B)** The *PtHMG1* relative expression of WT and PtHMG1-OE strains. Data are the average of three biological replicates with error bars indicating standard deviations. Asterisks indicate significant differences relative to WT (*P* < 0.05) in two-tailed Student’s *t*-test.

To investigate the function of *PtHMG1*, we initially constructed *PtHMG1* overexpression strains. Following three rounds of selection using zeocin, the screening plate for transforming resistance was shown in the **Supplementary Figure 4**. We successfully obtained two overexpression strains, designated as *PtHMG1*-OE1 and *PtHMG1*-OE2. PCR detection was conducted on genomic DNA of both mutant and wild-type (WT) strains using gene-specific primers *PtHMG1*-R and a plasmid universal primer pPha-T1-F. No bands were detected in the wild-type, while both mutant strains showed clear bands, confirming successful transformation (**Figure 2A**). Furthermore, we performed qRT-PCR to examine the changes in *PtHMG1* expression after overexpression. Compared to the wild-type, the expression of *PtHMG1* was significantly increased in both overexpression strains (**Figure 2B**), demonstrating a successful overexpression effect.

### 3.3 Generation of transgenic *P. tricornutum* with *PtHMG1* knockout

**Figure 3:**
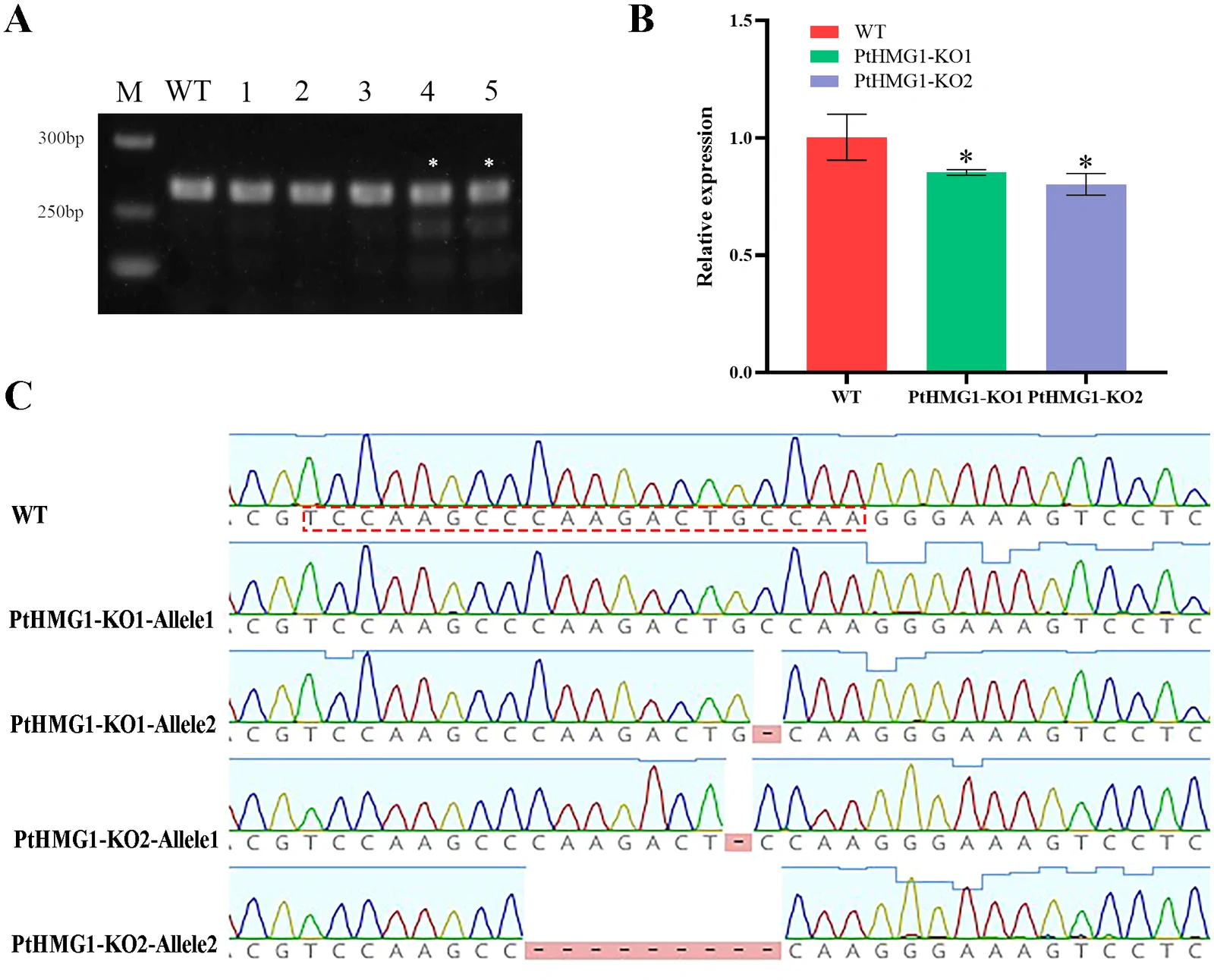
Generation of *PtHMG1* knockout (KO) strains by CRISPR/Cas9. **(A)** Example image of T7 endonuclease (T7EI) editing assay to screen exconjugants for potential editing events in the *PtHMG1*. Substrate indicates *PtHMG1* gene fragments amplified by PCR, while T7 product indicates exconjugants with evidence of Cas9 editing. M; 2 kb ladder with sizes indicated in basepairs (bp); WT is the wild-type algal strain genomic DNA and 1–5 are the different knockout algal strains genomic DNA used in the T7EI editing assay; asterisk represents editing success. **(B)** The *PtHMG1* relative expression of WT and PtHMG1-KO strains. Data values represent the average of three biological replicate, error bars represent SD. Asterisks indicate significant differences relative to WT (*P* < 0.05) in two-tailed Student’s *t*-test. **(C)** Example of mutagenic events through the alignment of the wild type, PtHMG1-KO1-Allele1, PtHMG1-KO1-Allele2, PtHMG1-KO2-Allele1 and PtHMG1-KO2-Allele2 subclones DNA sequences at the *PtHMG1* gene locus. The red dashed box indicates the sgRNA targeting positions 67–86 of the *PtHMG1* open reading frame.

To further elucidate the functional role of *PtHMG1* in fatty acid biosynthesis regulation, we generated two independent knockout lines using the CRISPR/Cas9 genome editing system. We first assessed editing by screening *P. tricornutum* exconjugants by T7 endonuclease I (T7EI) mismatch cleavage assays on PCR products amplified from the target gene (Wang et al., 2018). This assay identified 2 knockout strains with detectable editing rates based on screening of exconjugants (**Figure 3A**). We further verified the knockout effect using Sanger sequencing, and the results were shown in **Supplementary Figure 5**. The sequencing revealed the presence of mixed cell populations, a phenomenon referred to as mosaicism (Daboussi, 2017). After performing plate purification on the mosaicism, two colonies were sub-cloned to purity, verified by PCR and sequencing, and then stably maintained as *PtHMG1-*KO1 and *PtHMG1-*KO2. As expected, the two knockout algal strains had different mutations at the sgRNA binding site (**Figure 3C**). For the KO1 line, sequencing confirmed that it was a chimeric mutant. Allele 1 remained intact (wild-type), whereas Allele 2 sustained a single-nucleotide deletion (-1 bp), inducing a catastrophic frameshift mutation downstream. The KO2 line represented a bi-allelic mutant. Allele 1 suffered a single-nucleotide deletion (-1 bp) causing a frameshift mutation, while Allele 2 underwent a 9-base pair deletion (-9 bp), which led to a precise in-frame deletion of three amino acids. Mutations in both the KO1 and KO2 lines, which are located within the sgRNA-binding site (corresponding to amino acids 23–29), successfully disrupted the downstream functional HMG-box domain spanning amino acids 49 to 164. Furthermore, qRT-PCR analysis revealed that the expression level of *PtHMG1* in the knockout strains was significantly reduced compared to the wild-type strain (*P* < 0.05), indicating the effectiveness of the gene knockout (**Figure 3B**). Consequently, these two knockout algal strains *PtHMG1*-KO1 and *PtHMG1*-KO2 were utilized in subsequent experiments.

### 3.4 Functional characterization of *PtHMG1* in the growth regulation

**Figure 4:**
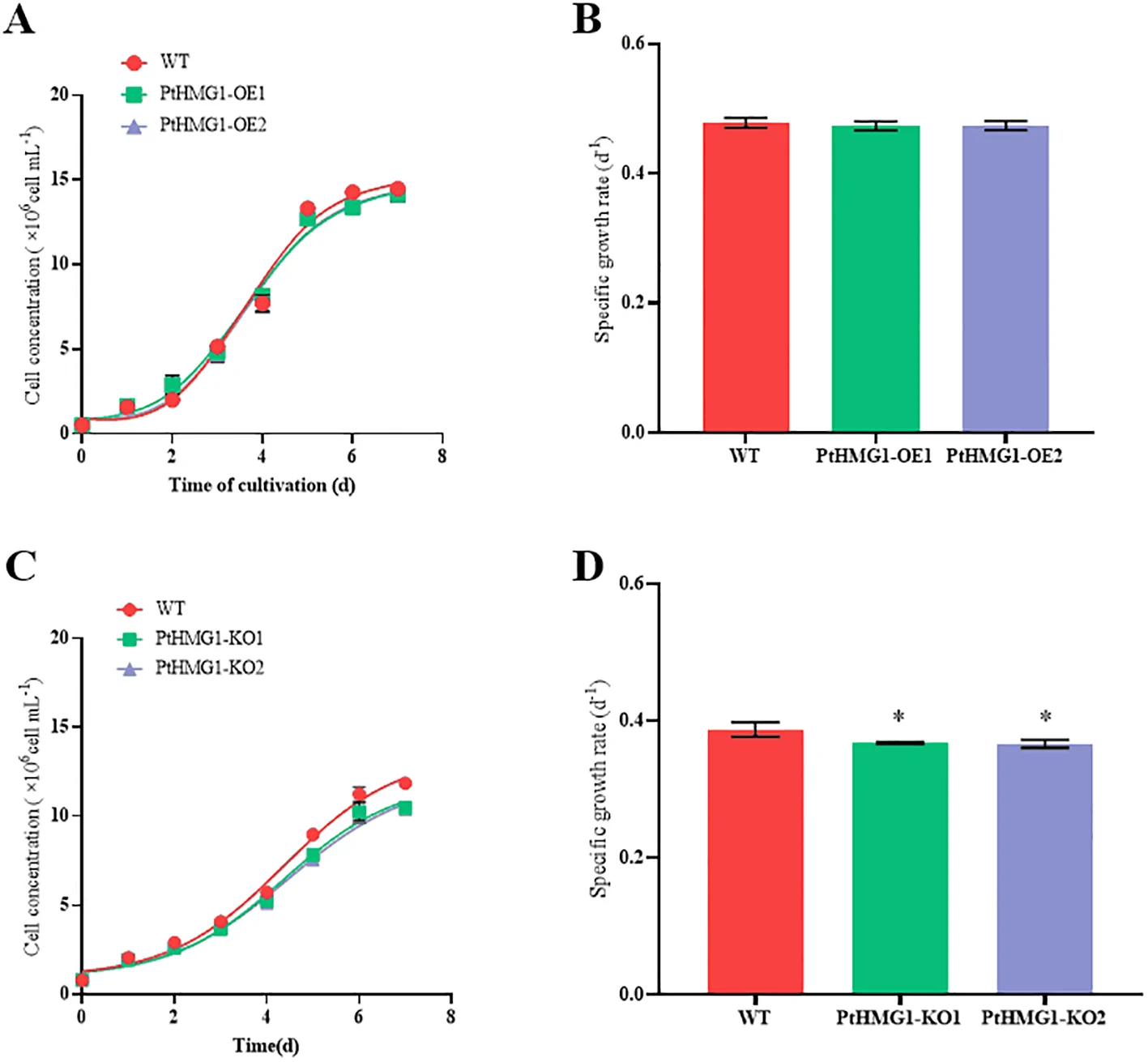
**(A)** Growth curves of WT and PtHMG1-OE strains. **(B)** Specific growth rates of WT and *PtHMG1*-OE strains. **(C)** Growth curves of WT and *PtHMG1*-KO strains. **(D)** Specific growth rates of WT and *PtHMG1*-KO strains. Data are the average of three biological replicates with error bars indicating standard deviations. Asterisk indicates statistically significant difference between a mutant and the WT during the respective condition (*P* < 0.05).

We investigated the regulatory role of *PtHMG1* on the growth of *P. tricornutum*. Our findings indicated that the *PtHMG1* overexpressing algal strain showed no significant impact on algal growth dynamics, as evidenced by overlapping growth curves (**Figure 4A**) and comparable specific growth rates (*P* > 0.05) between overexpression mutants and wild-type strains (**Figure 4B**). Knockout of *PtHMG1*, however, caused marked growth defects. *PtHMG1* knockout algal strains maintained temporal growth progression (**Figure 4C**) but showed 11.6% (KO1) or 12.4% (KO2) reduced biomass yield and 5.1% (KO1) or 5.5% (KO2) lower peak growth rate (*P* < 0.05) versus wild-type (**Figure 4D**). This phenotypic dichotomy indicates *PtHMG1* functions as a growth amplifier rather than a core cell cycle component, with its absence attenuating but not arresting cellular proliferation.

### 3.5 Transcriptional regulation of fatty acid desaturases by *PtHMG1*

**Figure 5:**
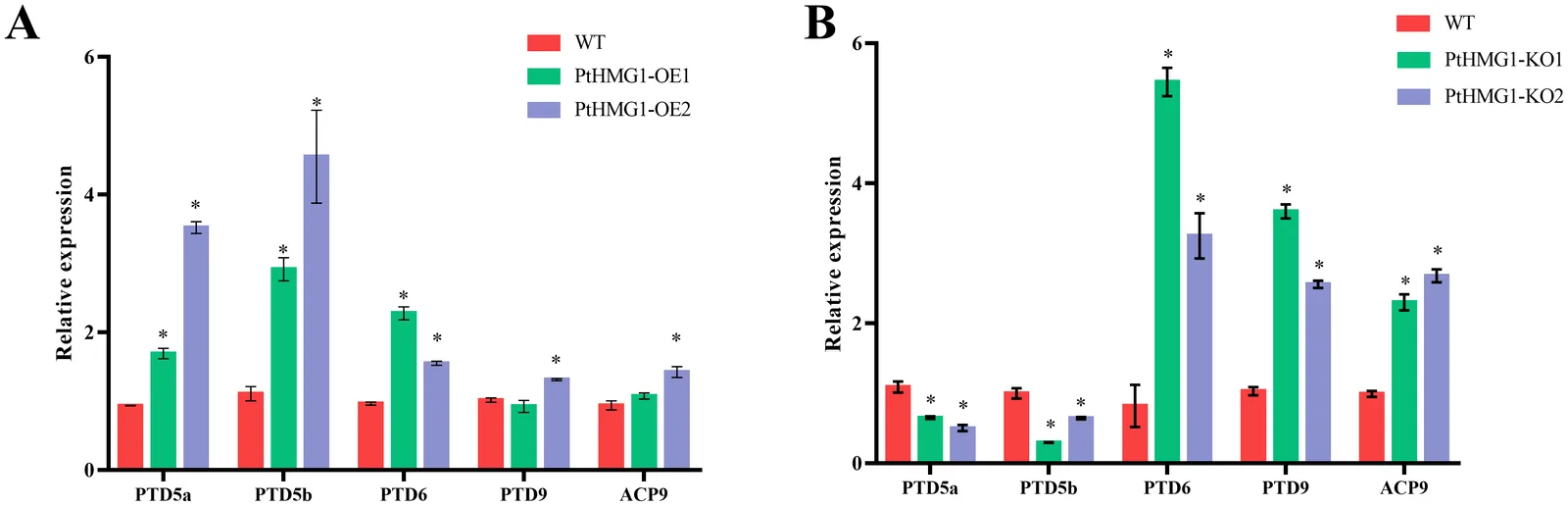
Effect of *PtHMG1* on the expression of fatty acid desaturase genes (*PTD5a, PTD5b, PTD6, PTD9, ACP9*). Comparison of fatty acid desaturase gene expression in WT versus **(A)** PtHMG1-OE strains and **(B)** PtHMG1-KO strains. Gene expression levels are presented as fold-change relative to the WT. Data are the average of three biological replicates with error bars indicating standard deviations. Asterisk indicates statistically significant difference between a mutant and the WT during the respective condition (*P* < 0.05).

Desaturases are key enzymes involved in the biosynthesis of unsaturated fatty acids. To thoroughly investigate the molecular mechanisms by which *PtHMG1* influences fatty acid synthesis, we assessed the expression levels of several key desaturase genes *PTD5a*, *PTD5b*, *PTD6*, *PTD9* and *ACP9* following the overexpression of *PtHMG1* in algal cells. Overexpression of *PtHMG1* induced significant transcriptional upregulation (*P* < 0.05) across multiple desaturase genes, with *PTD5a* and *PTD5b* demonstrating the most pronounced activation (2.8- and 3.2-fold increases, respectively) compared to wild-type controls (**Figure 5A**). Conversely, knockout of *PtHMG1* produced a divergent expression profile: *PTD5a/b* transcripts decreased by 65-72% (*P* < 0.05), while *PTD6*, *PTD9*, and *ACP9* exhibited compensatory upregulation relative to wild-type levels (**Figure 5B**). This reciprocal regulation pattern establishes *PtHMG1* as a positive transcriptional regulator of *PTD5a/b* paralogs, while its absence triggers pathway-specific compensatory mechanisms through alternative desaturase activation.

### 3.6 Effect of *PtHMG1* on lipid production

**Figure 6:**
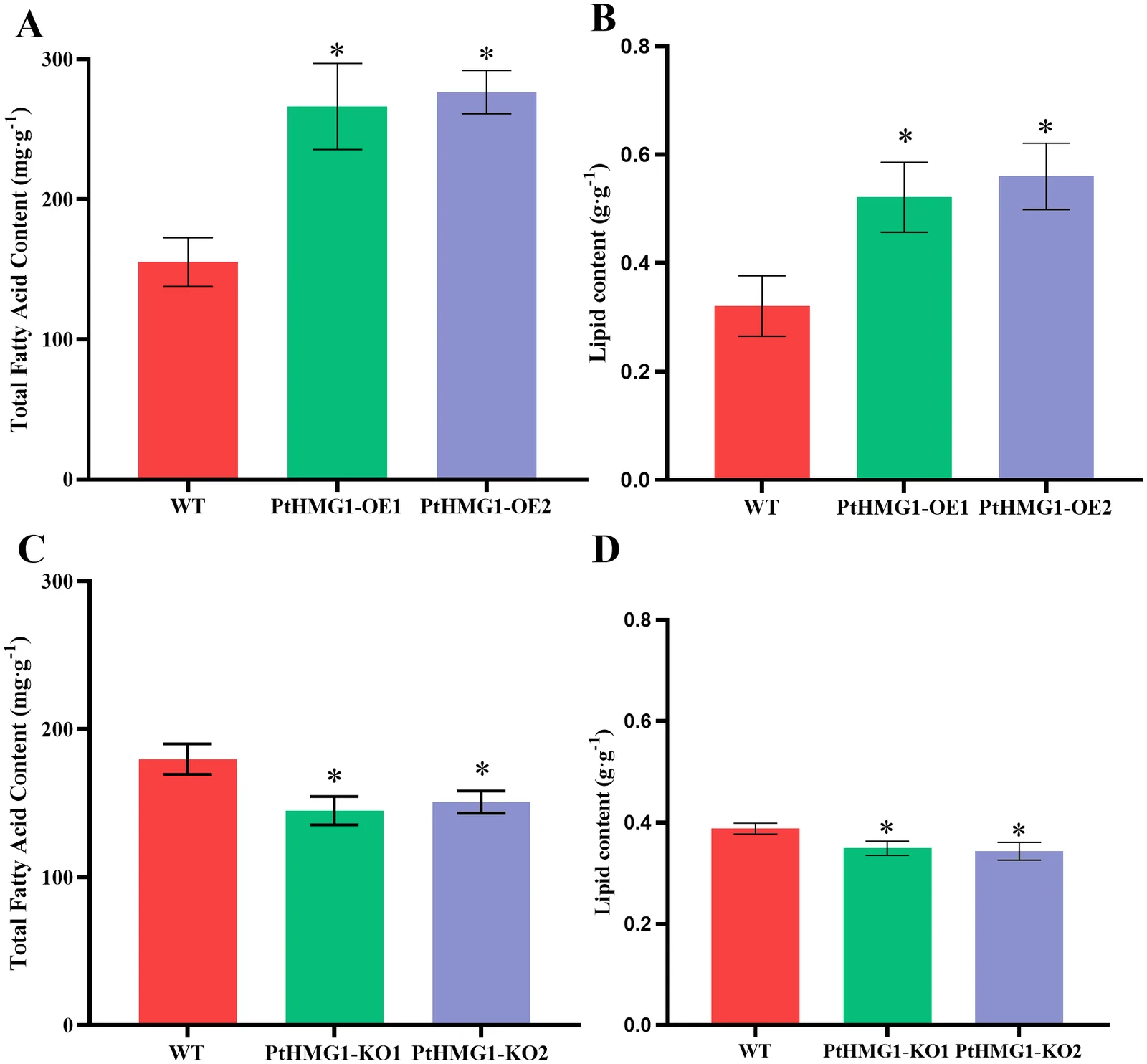
Total fatty acids (mg g^-1^DCW) and lipid content (g g^-1^DCW): comparison of WT and mutant strains on day 7. **(A)** The total fatty acid content of the WT and *PtHMG1*-OE strains. **(B)** Lipid content of the WT and *PtHMG1*-OE strains. **(C)** The total fatty acid content of the WT and *PtHMG1*-KO strains. **(D)** Lipid content of the WT and *PtHMG1*-KO strains. Data are the average of three biological replicates with error bars indicating standard deviations. Asterisk indicates statistically significant (*P* < 0.05) difference between a mutant and the WT during the respective condition.

Overexpression of *PtHMG1* resulted in a marked elevation of cellular lipid content, with total fatty acid content increasing by 90% and total lipid content rising from 0.3 g g^-1^ to 0.5 g g^-1^ DCW (66.7% increase) relative to the wild type (**Figures 6A, B**). Conversely, the *PtHMG1* knockout strain exhibited a notable decline in both fatty acid and total lipid content. Specifically, the total fatty acid content decreased by approximately 13%, while the total lipid content diminished by around 11% (**Figures 6C, D**).

### 3.7 Effect of *PtHMG1* on fatty acid synthesis

The changes in individual fatty acid components were further studied. In terms of the absolute content of each component of fatty acids, almost all fatty acid components showed an upward trend in the mutant after overexpression of *PtHMG1*, especially saturated fatty acid (SFA), C16:0 and monounsaturated fatty acid (MUFA), C16:1n-7, which increased by up to 177% and 148%, respectively. The absolute content of EPA also rose significantly, by approximately 18% (**Table 2**). In terms of relative content, the overexpression of the *PtHMG1* gene led to a marked increase in both SFAs and MUFAs, with the most pronounced increases observed in C16:0 and C16:1n-7, consistent with the trends in absolute content. Conversely, for PUFAs, such as EPA, DHA, and other HUFAs, the overexpression of the *PtHMG1* gene generally resulted in a downward trend in their relative contents, with the relative content of EPA decreasing by 42.72% (**Table 2**). However, unlike the overexpression of *PtHMG1*, alterations in each fatty acid component of the *PtHMG1* knockout algal strains exhibited no significant differences in relative contents, except for certain individual components, and there was no discernible trend in the levels of key fatty acids such as EPA (**Table 3**). Regarding absolute content, most fatty acid components demonstrated a decreasing trend as the total fatty acid content declined. This includes SFAs (C16:0 and C18:0), MUFAs (C18:1n-9), and PUFAs (EPA), all of which decreased significantly (**Table 3**).

**Table 2:** Effect of *PtHMG1* overexpression on the fatty acid absolute content (mg g^-1^ DCW) and relative content (% total fatty acids).

Fatty acids	Absolute content (mg g^-1^ DCW)			Relative content (% total fatty acids)		
	WT	PtHMG1-OE1	PtHMG1-OE2	WT	PtHMG1-OE1	PtHMG1-OE2
C14:0	8.66 ± 1.37	17.48 ± 1.65*	18.31 ± 0.98*	6.49 ± 0.03	7.38 ± 0.21*	7.40 ± 0.06*
C16:0	15.86 ± 2.55	49.62 ± 11.41*	58.08 ± 7.22*	11.89 ± 0.02	20.73 ± 2.20*	23.41 ± 1.74*
C16:1n-7	25.07 ± 4.02	70.20 ± 15.07*	70.80 ± 2.74*	18.79 ± 0.03	29.36 ± 2.55*	28.63 ± 0.53*
C16:2	2.43 ± 0.40	1.20 ± 0.09#	1.40 ± 0.05#	1.82 ± 0.03	0.51 ± 0.03#	0.57 ± 0.05#
C16:3n-4	0.99 ± 0.14	1.24 ± 0.10	0.80 ± 0.22	0.74 ± 0.01	0.53 ± 0.10#	0.33 ± 0.11#
C16:4n-1	5.63 ± 0.90	4.49 ± 0.42	4.48 ± 0.31	4.22 ± 0.04	1.92 ± 0.37#	1.82 ± 0.23#
C18:0	0.68 ± 0.12	1.38 ± 0.26*	1.94 ± 0.42*	0.51 ± 0.01	0.58 ± 0.04	0.78 ± 0.13*
C18:1n-9	10.55 ± 1.71	18.73 ± 2.30*	20.69 ± 2.34*	7.90 ± 0.01	7.89 ± 0.05	8.34 ± 0.51
C18:1n-7	1.20 ± 0.20	1.09 ± 1.20	1.20 ± 0.15	0.90 ± 0.02	0.46 ± 0.03#	0.48 ± 0.05#
C18:2n-6	3.22 ± 0.58	2.76 ± 0.28	2.87 ± 0.15	2.41 ± 0.04	1.11 ± 0.16#	1.16 ± 0.07#
C18:3n-6	0.19 ± 0.04	0.15 ± 0.01	0.15 ± 0.02	0.14 ± 0.01	0.07 ± 0.01#	0.06 ± 0.01#
C18:3n-3	0.12 ± 0.02	0.05 ± 0.01#	0.04 ± 0.01 #	0.09 ± 0.01	0.02 ± 0.01#	0.02 ± 0.01#
C18:4n-3	0.34 ± 0.06	0.48 ± 0.01*	0.44 ± 0.01*	0.25 ± 0.01	0.20 ± 0.02#	0.18 ± 0.01#
C20:3n-3	0.72 ± 0.12	2.55 ± 0.23*	1.96 ± 0.30*	0.54 ± 0.01	1.09 ± 0.22*	0.80 ± 0.17
C20:4n-6	0.10 ± 0.01	0.10 ± 0.01	0.07 ± 0.01	0.07 ± 0.01	0.04 ± 0.01#	0.03 ± 0.01#
C20:4n-3	0.37 ± 0.05	0.92 ± 0.02*	1.00 ± 0.21*	0.27 ± 0.01	0.39 ± 0.04*	0.40 ± 0.07*
C24:0	2.19 ± 0.36	2.32 ± 0.21	2.50 ± 0.04	1.64 ± 0.06	0.98 ± 0.04#	1.01 ± 0.06#
C20:5n-3	35.62 ± 2.50	42.44 ± 0.72*	41.30 ± 0.51*	29.20 ± 0.20	18.06 ± 2.20#	16.73 ± 0.93#
C22:5n-3	0.66 ± 0.09	0.49 ± 0.04	0.71 ± 0.04	0.50 ± 0.01	0.21 ± 0.04#	0.29 ± 0.03#
C22:6n-3	1.58 ± 0.25	1.09 ± 0.13	1.41 ± 0.08	1.19 ± 0.01	0.46 ± 0.01#	0.57 ± 0.01#
SFAs	27.39 ± 4.39	70.80 ± 13.60*	80.83 ± 8.66*	20.53 ± 0.06	32.61 ± 1.80*	29.67 ± 2.02*
MUFAs	36.83 ± 5.93	90.02 ± 18.57*	92.69 ± 5.23*	27.60 ± 0.03	37.46 ± 0.28*	37.72 ± 2.56*
PUFAs	51.97 ± 4.31	57.96 ± 2.07*	56.62 ± 1.92	41.45 ± 0.18	23.35 ± 1.32#	24.61 ± 3.17#
HUFAs	39.05 ± 2.75	47.59 ± 1.15*	46.45 ± 1.15*	31.77 ± 0.20	18.81 ± 1.06#	20.25 ± 2.50#

Data values represent the average of three biological replicates (mean ± SD). Significant differences relative to the WT (*P* < 0.05, two-tailed Student’s *t*-test) are indicated by asterisks (for increases) and octothorpes (for decreases).

**Table 3:** Effect of *PtHMG1* knockout on the fatty acid absolute content (mg g^-1^ DCW) and relative content (% total fatty acids).

Fatty acids	Absolute content (mg g^-1^DCW)			Relative content (% total fatty acids)		
	WT	PtHMG1-KO1	PtHMG1-KO2	WT	PtHMG1-KO1	PtHMG1-KO2
C14:0	10.84 ± 1.01	9.11 ± 0.84	9.26 ± 0.56	7.24 ± 0.20	7.53 ± 0.10	7.37 ± 0.12
C16:0	16.27 ± 1.29	12.23 ± 1.42#	13.21 ± 0.63#	10.87 ± 0.24	10.10 ± 0.53	10.52 ± 0.18
C16:1n-7	30.85 ± 1.69	26.37 ± 1.28	28.00 ± 3.01	20.63 ± 0.67	21.85 ± 0.76	22.25 ± 1.10
C16:2	0.39 ± 0.11	0.49 ± 0.03	0.49 ± 0.01	0.27 ± 0.08	0.40 ± 0.01*	0.39 ± 0.02*
C16:3n-4	0.66 ± 0.05	0.67 ± 0.04	0.75 ± 0.10	0.44 ± 0.03	0.55 ± 0.02*	0.59 ± 0.05*
C16:4n-1	6.34 ± 0.57	5.14 ± 0.54#	5.37 ± 0.40	4.23 ± 0.09	4.25 ± 0.12	4.27 ± 0.06
C18:0	0.67 ± 0.13	0.41 ± 0.01#	0.57 ± 0.06	0.45 ± 0.09	0.34 ± 0.04	0.45 ± 0.02
C18:1n-9	14.77 ± 0.86	11.79 ± 0.69#	12.06 ± 0.70#	9.88 ± 0.12	9.77 ± 0.26	9.60 ± 0.04
C18:1n-7	0.62 ± 0.02	0.50 ± 0.02#	0.58 ± 0.08	0.41 ± 0.01	0.41 ± 0.02	0.46 ± 0.04
C18:2n-6	3.93 ± 0.68	3.00 ± 0.50#	2.83 ± 0.34#	2.62 ± 0.38	2.48 ± 0.27	2.15 ± 0.08
C18:3n-6	0.47 ± 0.08	0.31 ± 0.04#	0.26 ± 0.02#	0.31 ± 0.04	0.25 ± 0.01	0.20 ± 0.02#
C18:3n-3	1.08 ± 0.11	0.70 ± 0.16#	0.74 ± 0.09#	0.72 ± 0.03	0.58 ± 0.10	0.59 ± 0.11
C18:4n-3	0.33 ± 0.01	0.27 ± 0.03#	0.28 ± 0.02	0.22 ± 0.01	0.22 ± 0.01	0.23 ± 0.00
C20:3n-3	1.81 ± 0.17	1.78 ± 0.23	1.90 ± 0.08	1.21 ± 0.10	1.47 ± 0.08*	1.51 ± 0.08*
C20:4n-6	0.07 ± 0.01	0.09 ± 0.01	0.09 ± 0.00	0.05 ± 0.00	0.08 ± 0.00*	0.07 ± 0.00*
C20:4n-3	0.49 ± 0.08	0.30 ± 0.11#	0.34 ± 0.14	0.32 ± 0.04	0.25 ± 0.09	0.27 ± 0.13
C24:0	2.03 ± 0.17	1.77 ± 0.22	1.82 ± 0.09	1.35 ± 0.02	1.46 ± 0.07	1.45 ± 0.06
C20:5n-3	41.19 ± 3.22	32.34 ± 2.95#	33.28 ± 1.34#	27.52 ± 0.29	26.73 ± 0.38	26.51 ± 0.60
C22:5n-3	0.72 ± 0.09	0.61 ± 0.13	0.68 ± 0.06	0.48 ± 0.03	0.50 ± 0.07	0.54 ± 0.08
C22:6n-3	0.97 ± 0.08	0.98 ± 0.08	0.98 ± 0.02	0.65 ± 0.02	0.81 ± 0.01*	0.78 ± 0.03*
SFAs	29.81 ± 2.50	23.52 ± 2.40#	24.86 ± 1.31	19.91 ± 0.49	19.44 ± 0.55	19.80 ± 0.31
MUFAs	46.23 ± 2.53	38.66 ± 1.99#	40.63 ± 3.76	30.92 ± 0.79	32.03 ± 1.04	32.31 ± 1.09
PUFAs	58.46 ± 4.69	46.69 ± 4.63#	47.98 ± 2.01#	39.05 ± 0.44	38.58 ± 0.81	38.12 ± 0.87
HUFAs	45.25 ± 3.57	36.11 ± 3.45#	37.26 ± 1.24#	31.58 ± 0.39	31.31 ± 0.61	31.15 ± 0.93

Data values represent the average of three biological replicate (mean ± SD). Significant differences relative to the WT (*P* < 0.05, two-tailed Student’s t-test) are indicated by asterisks (for increases) and octothorpes (for decreases).

## 4 Discussion

In this study, we characterized *PtHMG1* (ID: 7199075) as a regulatory factor that modulates both total lipid content and the fatty acid profile in *P. tricornutum*. Our empirical findings demonstrate that *PtHMG1* acts as a positive regulator of total fatty acid accumulation, while also significantly altering the downstream fatty acid composition. While previous studies, such as those by Sachdev et al. on *Arabidopsis* (Sachdev et al., 2024) and research on brown algae (Luthringer et al., 2024), have primarily linked HMG-box proteins to developmental transitions and sex determination, our work expands the functional scope of these regulators to microalgal lipid metabolism.

Our results show that while *PtHMG1* overexpression significantly boosted total lipid accumulation, it did not impose a noticeable metabolic burden on cellular proliferation (**Figures 4A, B**). In microalgal bioengineering, a common trade-off exists where diverted carbon flux toward lipid reservoirs occurs at the expense of biomass (Kwak et al., 2016; Dhokane et al., 2023; Song et al., 2023). The ability of *PtHMG1*-OE strains to decouple lipid hyper-accumulation from growth inhibition suggests that *PtHMG1* may enhance the overall efficiency of carbon fixation or partitioning, rather than simply shunting carbon from growth to storage.

Conversely, the significant reduction in biomass yield and growth rate in *PtHMG1*-KO strains (**Figures 4C, D**) underscores its importance for maintaining normal cell growth in *P. tricornutum.* The growth defects observed in the KO strains correlated with the downregulation of specific desaturases involved in EPA biosynthesis (**Figure 5**). Given that LC-PUFAs are essential components of diatom cellular membranes, the altered fatty acid profiles resulting from *PtHMG1* disruption may disrupt optimal membrane functionality and subsequent biomass accumulation (Li and Yu, 2018). Taken together, these results demonstrate that while the baseline expression of *PtHMG1* is required to support typical growth, its targeted upregulation holds substantial potential to enhance lipid accumulation without imposing significant negative impacts on cellular fitness.

A striking finding in our study is the distinct shift in the fatty acid profile observed in the *PtHMG1*-OE strain. Despite the significant upregulation of desaturases (*PTD5a*, *PTD5b*, *PTD6*) and a corresponding 18% increase in absolute EPA content, the relative proportion of PUFAs markedly declined (**Table 2**; **Figure 5A**). In *P. tricornutum*, C16:0-ACP synthesized *de novo* in the plastid represents a critical metabolic branch point. According to the established model of carbon partitioning in this diatom, 16:0-ACP can either be desaturated within the plastid by plastidial Delta9 desaturase (*PtPAD*) to form C16:1n-7-ACP (prokaryotic pathway), or exported to the cytosol as C16:0-CoA for subsequent elongation to C18 precursors in the endoplasmic reticulum—a prerequisite step for downstream EPA biosynthesis (eukaryotic pathway) (Arao et al., 1994; Li-Beisson et al., 2019). Consequently, the ratio of C16:1n-7 to EPA reflects the substrate competition for C16:0 between the prokaryotic and eukaryotic pathways, as evidenced by the phenotypes of *PtPAD* mutants (Smith et al., 2021). In the present study, *PtHMG1* overexpression led to a 74.35% and 96.89% increase in C16:0 percentage content compared with the wild type (**Table 2**), reflecting an enhanced substrate pool. Consequently, C16:1n-7 percentage levels in the prokaryotic pathway rose by 56.25% and 52.37%, while C18:1n-9 in the eukaryotic pathway remained unaffected, leading to a concomitant decrease in the relative proportion of the eukaryotic end-product EPA. We speculate that the eukaryotic PUFA pathway, which is longer and encompasses further rate-limiting bottlenecks like elongation (Hamilton et al., 2014), could not effectively compete with the prokaryotic pathway for the shared C16:0 substrate despite the generalized upregulation of desaturases, ultimately diluting the relative EPA accumulation.

In contrast, the *PtHMG1*-KO phenotype illustrates the regulatory response of *P. tricornutum* to the disruption of this factor. The 11% reduction in total lipids and the targeted downregulation of front-end desaturases (*PTD5a/b*) directly account for the decrease in absolute EPA content. However, the relative proportions of the remaining fatty acids remained remarkably stable (**Table 3**). This reflects a dynamic homeostatic regulation of the cellular fatty acid composition in response to both the reduction of the end-product EPA and the constrained supply of the initial substrate C16:0.

Based on the above analysis, we propose that *PtHMG1* coordinates the fatty acid biosynthetic pathway through both transcriptional control and end-product feedback loops. On one hand, *PtHMG1* promotes *de novo* fatty acid synthesis to generate core substrates like C16:0 (**Table 2**), while also positively regulating the terminal desaturases *PTD5a* and *PTD5b* (**Figure 5A**). In the PtHMG1-OE lines, the simultaneous increase in upstream substrate supply and terminal enzyme expression efficiently channels the carbon flux toward EPA accumulation. On the other hand, the disruption of *PtHMG1* creates a dual deficit of both the initial substrate C16:0 and the end-product EPA (**Table 3**). In the KO strains, the loss of positive regulation by *PtHMG1* leads to the downregulation of *PTD5a* and *PTD5b*, directly restricting EPA synthesis (**Figure 5B**). Crucially, the resulting depletion of the end-product (EPA) relieves the negative feedback inhibition that normally suppresses the intermediate desaturases (*PTD6*, *PTD9* and *ACP9*), as has been widely reported in other organisms (Njoroge et al., 2012; Czumaj and Śledziński, 2020; Monirujjaman et al., 2023). This feedback relief explains the paradoxical upregulation of these intermediate genes (**Figure 5B**). However, because the upstream *de novo* supply remains blocked in the KO strains, this transcriptional increase does not lead to actual lipid accumulation, as shown by the unchanged fatty acid profiles (**Table 3**). Nevertheless, further research is needed to determine whether *PtHMG1* controls *de novo* fatty acid biosynthesis and the expression of PTD5a and PTD5b directly or through the recruitment of other transcription factors.

Taken together, our results indicate that *PtHMG1* plays a substantial role in modulating both the lipid accumulation and the overall fatty acid profile in *P. tricornutum*. These data suggest that *PtHMG1* expression is strongly linked to the capacity for high-yield lipid production, and its further characterization underscores its potential as a genetic target to optimize lipid productivity and composition in diatoms. Given the structural flexibility typical of HMG-box proteins, it may modulate the expression of multiple lipid and fatty acid biosynthetic genes by dynamically recruiting specific co-factors or chromatin-remodeling complexes. However, the precise molecular interactions and direct downstream targets of *PtHMG1* remain to be fully elucidated. Future investigations will be essential to map its genome-wide binding landscape and identify its coregulatory partners, thereby uncovering the underlying genetic mechanisms governing microalgal lipid homeostasis.

## 5 Conclusion

This study characterizes *PtHMG1* as a key transcriptional regulator that modulates lipid accumulation and fatty acid profiles in the model diatom *P. tricornutum*. By integrating phenotypic and expression data from both overexpression and CRISPR/Cas9-mediated knockout strains, we demonstrate that *PtHMG1* plays a substantial role in regulating fatty acid composition rather than acting as a simple binary switch. Our findings reveal that while *PtHMG1* expression is a potent driver of total lipid yields—nearly doubling fatty acid accumulation in overexpression strains—it inherently impacts the relative balance among different fatty acid classes. Although *PtHMG1* upregulation increases absolute EPA content, the concurrent and substantial rise in saturated and monounsaturated fatty acid synthesis leads to a relative decrease in the proportion of polyunsaturated fatty acids (PUFAs). Conversely, the stability of fatty acid relative proportions in knockout strains suggests robust transcriptional feedback aimed at maintaining cellular homeostasis when *PtHMG1* is lost. From a biotechnological perspective, this research highlights *PtHMG1* as a promising genetic target for targeted metabolic engineering to optimize microalgal oil production and provides a sound theoretical foundation for developing high-yield diatom strains.

## Data Availability

The original contributions presented in the study are included in the article/**Supplementary Material**, further inquiries can be directed to the corresponding author/s.
