# Supplementary material for: The HMG-box protein PtHMG1 modulates lipid accumulation and fatty acid composition in the diatom Phaeodactylum tricornutum
Source: Front Plant Sci. 2026 Aug 6;17:1863846. doi: 10.3389/fpls.2026.1863846 (PMC13490174; doi:10.3389/fpls.2026.1863846)
Supplement: Supplementary file 1 [file DataSheet1.docx]

Supplementary Material

# 1 Supplementary Figures and Tables

## 1.1 Supplementary Figures

**Supplementary Figure 1.** The schematic diagram of plasmid vector pPha-T1-PtHMG1.

**Supplementary Figure 2.** The schematic diagram of Crisper/Cas9 plasmid vector pPtGE35.

**Supplementary Figure 3.** Multiple sequence alignment of representative HMG-box domains from *Phaeodactylum tricornutum* (Pt), *Chlamydomonas reinhardtii* (Cr), *Saccharomyces cerevisiae* (Sc)，*Arabidopsis thaliana* (At), *Oryza sativa* (Os), and *Homo sapiens* (Hs). Strictly identical residues are highlighted with a white character on a red background, while highly similar residues are clustered in blue frames and written in red.

**Supplementary Figure 4.** Screening of the transformation-resistant plates. The left plate is the blasticidin-resistant plate with the unsuccessful transformation, and the right plate is the blasticidin-resistant plate containing the successful transformants.

**Supplementary Figure 5.** Sequence chromatogram of a PCR product amplified from knockout strains, showing the presence of heterogeneous sequences surrounding the cutting site.

## 1.2 Supplementary Tables

**Tab.S1** Sequences of primer pairs used in this study.

| **Gene ID** | **Gene Name** | **Abbreviation** | **Primers** **(5’-3’)** | **Application** |
| --- | --- | --- | --- | --- |
| PHATRDRAFT_50336 (XM_002185227.1) | PtHMG1 | PtHMG1 | PtHMG1-F: caggtcgactctagaggatccATGACTAGTTACGACGCTGTGGG | Homologous recombinant plasmid constructs |
|  |  |  | PtHMG1-R: tgtctgccgtttcgagaattcTCACTTGTTTTGATCGTCCTGTTG |  |
| PHATRDRAFT_50336  (XM_002185227.1) | PtHMG1 | PtHMG1 | PtHMG1-qPCR-F: ATGACTAGTTACGACGCTGTGG | RT-qPCR of PtHMG1 gene |
|  |  |  | PtHMG1-qPCR-R: CCCACCAATCATCTGGTCATTC |  |
|  | pPha-T1 Plasmid | pPha-T1 | pPha-T1-F: CTTCTTCGAGTCCTGTTAC | pPha-T1 universal primers |
|  |  |  | pPha-T1-R: GAAGGATAGAGACTCCCAAC |  |
| PHATRDRAFT_51157  (XM_002183388.1) | ACT1 | Actin | Actin-F: CTTCTTCGAGTCCTGTTAC | For RT-qPCR |
|  |  |  | Actin-R: GACCCTCCAATCCAAACAGAG |  |
| PHATRDRAFT_9316  (XM_002177381.1) | stearoyl-ACP desaturase | ACP9 | ACP9-F: GCAGAAATCATCGACCATCTGG | For RT-qPCR |
|  |  |  | ACP9-R: TCAAGCCATCCGACCATAAATCC |  |
| PHATR_46830  (XM_002185696.1) | The Delta5 Fatty Acid Desaturase | PTD5a | PTD5a-F: GTTCTTCCGTGCGTTTTGC | For RT-qPCR |
|  |  |  | PTD5a-R: TGGTGTAAGCGTGGTGGGT |  |
| PHATRDRAFT_22459  (XM_002182822.1) | The Delta5 Fatty Acid Desaturase | PTD5b | PTD5b-F: GCACCGTGGGTTACTTCTT | For RT-qPCR |
|  |  |  | PTD5b-R: ATTTACAGCCACCAATGAGAT |  |
| PHATRDRAFT_29488  (XM_002182865.1) | The Delta6 Fatty Acid Desaturase | PTD6 | PTD6-F: GGCAGGAAGTCAAGACCCAC | For RT-qPCR |
|  |  |  | PTD6-R: GCAGCAATTCGCCAATGTAG |  |
| PHATRDRAFT_28797  (XM_002181758.1) | The Delta9 Fatty Acid Desaturase  (stearoyl-CoA desaturase) | PTD9 | PTD9-F: TTCTTTGCGCACATGGGTTG | For RT-qPCR |
|  |  |  | PTD9-R: ACTTGTGCCGGCATGACATA |  |
|  |  | sgRNA | sgRNA-87- F: **TCGA**TCCAAGCCCAAGACTGCCAA | For Crisper/cas9 |
|  |  |  | sgRNA-87-R: **AAAC**TTGGCAGTCTTGGGCTTGGA |  |
|  | pPtGE35 Plasmid | pPtGE35 | pPtGE35-F: GTTGCAGTCACTCCGCTTTG | pPtGE35 universal primers |
|  |  |  | pPtGE35-R: GCAGAAAAGTTCGTCGAGCC |  |
|  |  | PtHMG1-T7 | PtHMG1-T7-F: TTCCCTTTTGTAGACGGGGC | For T7 endonuclease detection |
|  |  |  | PtHMG1-T7-R: TGCTTCCGCGAACGATATGA |  |
